# Transcranial Direct Current Stimulation Alleviates Neurovascular Unit Dysfunction in Mice With Preclinical Alzheimer’s Disease

**DOI:** 10.3389/fnagi.2022.857415

**Published:** 2022-04-14

**Authors:** Yinpei Luo, Hong Yang, Xiaojing Yan, Yaran Wu, Guoliang Wei, Xiaoying Wu, Xuelong Tian, Ying Xiong, Guangyan Wu, Huizhong Wen

**Affiliations:** ^1^Chongqing Key Laboratory of Neurobiology, Department of Neurobiology, Army Medical University, Chongqing, China; ^2^Laboratory of Neural Regulation and Rehabilitation Technology, Chongqing Medical Electronics Engineering Technology Research Center, College of Bioengineering, Chongqing University, Chongqing, China; ^3^Department of Biochemistry and Molecular Biology, Army Medical University, Chongqing, China; ^4^Experimental Center of Basic Medicine, Army Medical University, Chongqing, China

**Keywords:** transcranial direct current stimulation, Alzheimer’s disease, neurovascular unit, amyloid-β, astrocyte, blood vessel, blood-brain barrier

## Abstract

Neurons, glial cells and blood vessels are collectively referred to as the neurovascular unit (NVU). In the Alzheimer’s disease (AD) brain, the main components of the NVU undergo pathological changes. Transcranial direct current stimulation (tDCS) can protect neurons, induce changes in glial cells, regulate cerebral blood flow, and exert long-term neuroprotection. However, the mechanism by which tDCS improves NVU function is unclear. In this study, we explored the effect of tDCS on the NVU in mice with preclinical AD and the related mechanisms. 10 sessions of tDCS were given to six-month-old male APP/PS1 mice in the preclinical stage. The model group, sham stimulation group, and control group were made up of APP/PS1 mice and C57 mice of the same age. All mice were histologically evaluated two months after receiving tDCS. Protein content was measured using Western blotting and an enzyme-linked immunosorbent assay (ELISA). The link between glial cells and blood vessels was studied using immunofluorescence staining and lectin staining. The results showed that tDCS affected the metabolism of Aβ; the levels of Aβ, amyloid precursor protein (APP) and BACE1 were significantly reduced, and the levels of ADAM10 were significantly increased in the frontal cortex and hippocampus in the stimulation group. In the stimulation group, tDCS reduced the protein levels of Iba1 and GFAP and increased the protein levels of NeuN, LRP1 and PDGRFβ. This suggests that tDCS can improve NVU function in APP/PS1 mice in the preclinical stage. Increased blood vessel density and blood vessel length, decreased IgG extravasation, and increased the protein levels of occludin and coverage of astrocyte foot processes with blood vessels suggested that tDCS had a protective effect on the blood-brain barrier. Furthermore, the increased numbers of Vimentin, S100 expression and blood vessels (lectin-positive) around Aβ indicated that the effect of tDCS was mediated by astrocytes and blood vessels. There was no significant difference in these parameters between the model group and the sham stimulation group. In conclusion, our results show that tDCS can improve NVU function in APP/PS1 mice in the preclinical stage, providing further support for the use of tDCS as a treatment for AD.

## Introduction

Alzheimer’s disease (AD) is the most common dementia in the world, and its main clinical manifestations are progressive cognitive impairment and memory decline. Its high prevalence, morbidity, mortality and care costs have brought a huge burden to the society (Alzheimer’s Association, 2021). The main pathological features of AD are amyloid-β (Aβ) protein aggregation and tau protein hyperphosphorylation (Alzheimer’s Association, 2021). These changes are accompanied by neuronal degeneration, neuroinflammation, and damage to the blood-brain barrier. Neurons, glial cells and cerebral blood vessels are closely related in structure and function, collectively referred to as neurovascular unit (NVU), which is very important for regulating cerebral blood flow, maintaining the blood-brain barrier and signal transduction between cells (Liu et al., 2019; Stackhouse and Mishra, 2021). According to the two-hit hypothesis of AD, NVU dysfunction and the imbalance of Aβ production and degradation accelerate the process of AD (Zlokovic, 2005; Cai et al., 2017). Aging can impair the structure and function of the different components of NVU (Wei et al., 2016). There is evidence that in the AD brain, the cerebral microcirculation system is damaged, and the main components of NVU have undergone pathological changes (Kirabali et al., 2020). In recent years, reducing the unfavorable factors of NVU has become a potential treatment direction for AD. However, it is still unclear whether improving the pathological state of NVU can improve the symptoms of AD, the relationship between the pathological changes of NVU and the pathology of AD. One possible factor is related to astrocyte dysfunction.

Astrocytes, the main glial cells, are highly differentiated cells in the central nervous system. Astrocyte endfeet surround more than 99% of the cerebral blood vessel surface (Iadecola and Nedergaard, 2007). Astrocytes are interconnected with cerebral blood vessels and neurons (Price et al., 2021), which can promote the uptake and synthesis of neurotransmitters, induce angiogenesis and regulate cerebral blood flow, thus contributing to neuroprotection and repair of damaged tissues (Abbott et al., 2006; Sofroniew and Vinters, 2010). However, in AD, reactive astrocytes may also secrete vascular permeability factors to destroy the blood-brain barrier, produce proinflammatory factors to cause neuroinflammation and induce an inflammatory cascade, impair Aβ clearance, and ultimately aggravate Aβ accumulation and neuronal degeneration (Bush et al., 1999; Zhao et al., 2021). A recent study found that the integrity of blood vessels can be maintained in the absence of astrocyte endfeet (Kubotera et al., 2019). This finding further complicates the relationship between astrocytes and blood vessels. Pathological changes of astrocytes and blood vessels are believed to start 20 years or earlier before the clinical symptoms of AD (Barthelemy et al., 2020; Quiroz et al., 2020). It is worth exploring whether it is possible to slow the progression of AD by promoting the interaction between astrocytes and blood vessels in the preclinical stage of AD.

Transcranial direct current stimulation (tDCS) is a non-invasive neuromodulatory technique that delivers direct current to the scalp above the target area by electrodes to alter the excitability of the cortex (Lefaucheur et al., 2017). Transcranial direct current stimulation has been found to improve cognition and slow the progression of the disease in human and animal models of AD (Yang et al., 2019; Saxena and Pal, 2021). According to reports, tDCS can protect neurons, induce changes in glial cells, and regulate cerebral blood flow (Hansen, 2012). In mice with preclinical AD, tDCS can improve learning and memory, reduce the number of astrocytes, and increase cerebral blood flow and blood flow velocity (Luo et al., 2020a,b). However, few studies have involved tDCS and NVU in preclinical AD. Therefore, it is worth investigating whether tDCS administration in preclinical AD can alleviate disease pathology by ameliorating the changes in the NVU.

This study aims to explore the effect of tDCS on the NVU in preclinical AD and the underlying mechanism related to astrocytes and blood vessels, and to provide reference for the application of tDCS in preclinical AD.

## Materials and Methods

### Animals

All animal experimental procedures performed in this study were approved by the Experimental Animal Welfare and Ethics Committee of Army Medical University. 33 male B6/J-Tg (APPswe/PSEN1dE9, APP/PS1) mice and 11 male C57BL/6J mice were purchased from Nanjing Junke Biotechnology Co., Ltd. (Nanjing, China). They were housed in a specific pathogen-free environment at the Experimental Animal Center of Army Medical University (Chongqing, China). The mice were housed 3∼4 per cage on a 12-h light/dark cycle and given sufficient food and water. 33 6-month-old male APP/PS1 double transgenic mice (weighing 25∼331 g) were randomly divided into 3 groups with 11 mice in each group: the tDCS treatment group (ADT group), sham stimulation group (ADS group) and unstimulated control group (AD group). 11 male C57BL/6J mice of the same age (weighing 26∼31 g) were used as the normal control group (WT group).

### Surgery and Transcranial Direct Current Stimulation Treatment

On the day before the formal experiment, all mice in the ADS group and the ADT group underwent anode electrode implantation as described previously (Yang et al., 2019). The mice were anesthetized with isoflurane (anesthetic concentration: 2∼3 L/min, air flow meter: 0. 3∼0.5 L/min, Shenzhen Reward Life Science Co., Ltd., Shenzhen, China), and then fixed in a stereotaxic frame to with low-concentration anesthetics via inhalation for anesthesia maintenance (anesthetic concentration: 1∼1.5 L/min, air flow meter: 0. 3∼0.5 L/min). The anode electrode was implanted on the skulls of the mice above the frontal cortex and fixed with non-toxic dental cement. The mice were then allowed to recover on a heating pad at a temperature of 24°C and returned to their home cages. Transcranial direct current stimulation treatment was carried out 24 h after the mice recovered. Before stimulation, a cathode reference electrode was attached to the chest and abdomen of the mice, and a head anode electrode was soaked in 0.9% NaCl solution to reduce the contact impedance. Mice in the ADT group were treated with tDCS for 30 min every day at a current intensity of 150 μA. A multimeter was used to monitor the real-time current delivered to the mice during stimulation. Mice in the ADS group received sham stimulation with 150 μA tDCS for only 10 s every day. tDCS treatment was administered on a cycle of 5 days of treatment and 2 days of rest. A total of 2 cycles were carried out. All mice were unrestrained during the stimulation period.

### Western Blotting

Three mice from each group were deeply anesthetized with isoflurane (anesthetic concentration: 2∼3 L/min, air flow meter: 0.3∼0.5 L/min) two months after the end of tDCS treatment, and then frontal cortex and hippocampal tissues were quickly removed and placed on ice. The tissues were washed with pre-cooled phosphate-buffered saline (PBS, 0.01 M, pH = 7.4) to remove the residual blood. Radioimmunoprecipitation assay lysis buffer (P0013B, Beyotime, Shanghai, China) containing phenylmethanesulfonyl fluoride protease inhibitor (ST506, Beyotime) was added to the tissues, the tissues were completely homogenized on ice with a grinder, the ground tissue homogenates were centrifuged in a low-temperature high-speed centrifuge at 8000 × g for 15 min, and the supernatant was collected. After the protein concentration in the supernatant was measured, 25 μg protein was diluted in buffer and ddH_2_O to a volume of 5 μl. After the samples were loaded, they were electrophoresed at a constant voltage of 70 V for 100 min and then transferred onto polyvinylidene fluoride membranes at a constant current of 100 mA in an icebox. After transfer, the membrane was removed and non-specific antigens were blocked with rapid blocking solution (P0252, Beyotime) on a shaking table at room temperature for 15 min. Membranes were then incubated with primary antibody (1:1000) overnight at 4°C. β-actin was used as an internal reference protein. After these membranes were removed the next day, it was rinsed with Tris-buffered saline with Tween 20 (TBST) on a shaking table at room temperature 5 times for 8 min each. The corresponding secondary antibody was added, and the membrane was incubated at 37°C for 1. 5 h. Then, these membranes were rinsed with TBST buffer 5 times for 8 min each at room temperature. The membranes were developed with chemiluminescent HRP substrate (WBKLS0100, Merck KGaA, Darmstadt, Germany) for 1 min and then placed in a gel imager (Bio-Rad Laboratories, Inc., California, United States) for imaging. The gray value of each membrane was measured with ImageJ software and normalized to the gray value of β-actin. Each experiment was repeated at least three times. The antibody information involved is shown in Table 1.

**TABLE 1:** Key resources table.

Type	Target	Host	Source	Catalog number	Dilution (WB)	Dilution (IF)
First antibody	Aβ	Mouse	Santa Cruz Biotechnology, Dallas, USA	SC-28365	–	1:1000
	NeuN	Rabbit	Merck, Darmstadt, Germany	ABN78	1:1000	–
	Iba1	Rabbit	ABclonal, Wuhan, China	A19776	1:1000	–
	LRP1	Rabbit	ABclonal	A0633	1:1000	–
	GFAP	Rabbit	Cell Signaling Technology, Danvers, MA, USA	80788	1:1000	1:1000
	PDGRFβ	Rabbit	Thermo Fisher, Shanghai, China	MA5-15143	1:1000	–
	β-actin	Rabbit	ABclonal	AC026	1:100000	–
	AQP4	Mouse	Santa Cruz, Dallas, TX, United States	sc-32739	–	1:1000
	Occludin	Rabbit	Abcam, Cambridge, United Kingdom	ab167161	–	1:1000
	S100β	Rabbit	Cell Signaling Technology	90393	–	1:1000
	Vimentin	Rabbit	ABclonal	A19607	–	1:200
Second antibody	Goat anti-mouse (H + L)	–	Zhongshan Goldenbridge Biotechnology, Beijing, China	ZB2305	–	1:2000
	Goat anti-rabbit (H + L)	–	Zhongshan Goldenbridge Biotechnology	ZB2301	–	1:2000
	Anti-mouse IgG Fab2, Alexa Fluor 488	Goat	Cell Signaling Technology	4408S	–	1:2000
	Anti-rabbit IgG Fab2, Alexa Fluor 647	Goat	Cell Signaling Technology	4414S	–	1:2000
	Anti-mouse IgG H&L, Alexa Fluor 594	Donkey	Abcam	ab150108	–	1:600

### Enzyme-Linked Immunosorbent Assay

Samples were obtained for ELISA in the same manner as for WB. Then, PBS containing protease inhibitors (P1005, Beyotime) was added to the tissues at a ratio of 1:9. A grinder was used to fully homogenize the tissues on ice, the samples were centrifuged at 8000 × *g* for 10 min in a low-temperature high-speed centrifuge, and the supernatant was collected. ELISA kits Mouse Aβ ELISA kit, mouse amyloid precursor protein (APP) ELISA kit, mouse β-site amyloid precursor protein-cleaving enzyme 1 (β-secretase; BACE1) ELISA kit, and mouse a disintegrin and metalloprotease domain 10 (ADAM10) ELISA kit) (Shanghai Enzyme-linked Biotechnology Co., Ltd., Shanghai, China) were placed at room temperature for 60 min. The test plate was taken out and 50 μl of different concentrations of the standard was added to the standard holes. Then, 50 μl of each sample to be tested was added to the sample holes, and 50 μl of PBS was added to the blank hole. Then, 100 μl of horseradish peroxidase-labeled antibody was added to the standard wells, sample wells, and blank wells. The plate was sealed with sealing film and incubated at 37°C for 60 min. The liquid was discarded, the plate was dried on absorbent paper, and each well was filled with wash solution (350 μl). The plate was incubated for 1 min, the wash solution was discarded, the plate was dried on absorbent paper, and the washing step was repeated 5 times. After washing, a substrate (100 μl) was added to each well. The plate was incubated in the dark at 37°C for 15 min, and then 50 μl of termination solution was added to each well. A microplate reader (Imark, Bio-Rad Laboratories, Inc. Hercules, California, United States) was used to measure the optical density of each well at a wavelength of 450 nm, and the measurement was repeated three times.

### Immunofluorescence Staining

Two months after tDCS treatment, the mice were deeply anesthetized with isoflurane (anesthetic concentration: 2∼3 L/min, air flow meter: 0.3∼0.5 L/min) and transcardially perfused with preheated NaCl (37°C) and 4% paraformaldehyde (PFA, 4°C). The brains were fixed with 4% PFA (4°C) overnight and then dehydrated with a gradient of 10%, 20% and 30% PFA sucrose solutions. The frontal cortex and hippocampal tissues were sectioned at a thickness of 30 μm with a freezing microtome (CM1900, Leica, MN, United States) for immunofluorescence staining. Brain slices containing the target area were rinsed three times with PBS (1%) for 10 min each time and then incubated with 10% goat serum (ZLI-9021, Zhongshan Biotech, Beijing, China) for 1 h in a 37°C incubator. The sections were incubated with the primary antibody in a 37°C incubator for 1 h and then incubated at 4°C overnight. The brain slices were removed and rinsed three times with 1% Tween 20 in PBS (PBST) for 10 min each. Then, the sections were incubated with fluorescent secondary antibody and tomato lectin (1:400, a marker of the vascular endothelium, DL-1177, Vector Laboratories, San Francisco Bay, United States) in a 37°C incubator for 1 h in the dark followed by 4’,6-diamidino-2-phenylindole (DAPI, 1:2000, D8417, Sigma-Aldrich, Poole, United Kingdom) for 10 min in the dark and rinsed three times with 1% PBST for 10 min each. The sections were sealed with Fluoromount-G fluorescent mounting medium (0100-01, Southern Biotech, Birmingham, AL, United States) and stored in a cassette. Endogenous immunoglobulin (IgG) staining was performed as previously described (Bonetti et al., 2021), and sections were washed 3 times in PBS and then incubated in donkey anti-mouse IgG H&L at 4°C for 24 h. Images (10x magnification) were acquired with Olympus SLIDEVIEW VS200 research slide scanner (Olympus, Japan) and Zeiss BX53 epifluorescence microscope (Zeiss, Jena, Germany) through Olympus cellSens Standard 1.16 software. High magnification fluorescent images (20x magnification) were acquired with Zeiss Axio Observer Z1/7 inverted epifluorescence microscope through Zen 2.3 software. The antibody information is shown in Table 1.

The area fraction and number of Aβ were used to evaluate the change of Aβ. Among them, the area fraction is the percentage of Aβ-positive expression area in the visual field. Vessel morphology was assessed by blood vessel density and blood vessel length. Blood vessel density was calculated by the area fraction of lectin-positive areas in the area. Blood vessel length was measured using the “Measuring Skeletal Length” plugin of ImageJ (Kirabali et al., 2020; Rust et al., 2020). IgG penetration, endothelial cell-specific junction protein, astrocyte and blood vessels to assess blood-brain barrier integrity. IgG extravasation was represented by the area fraction of the positive expression area in the field, a quantifiable indication of blood-brain barrier permeability (Bonetti et al., 2021). Colocalization between aquaporin 4 (AQP4, a marker of astrocyte foot processes) and lectin was calculated using Mander’s colocalization coefficient to assess astrocyte and vascular coverage (Duncombe et al., 2017). Fluorescence intensity was used to assess the expression levels of occludin (A marker of endothelial cell-specific junction proteins) and different astrocyte markers. ImageJ software was used to measure the above parameters.

### Statistical Analysis

The data were expressed as the mean ± standard error of the mean (SEM) and were analyzed using IBM SPSS statistics 26. When the observed values for each group were homoscedastic, the statistical significance of the difference between groups was analyzed by one-way analysis of variance followed by Tukey’s *post hoc* test. GraphPad Prism 8 was used to generate graphs. P < 0.05 was considered a significant difference, and significance was indicated as follows: *P* <0.05, */#; *P* <0.005, **/##; and *P* <0.001, ***/###. Specific statistical parameters were presented in the legends unless otherwise specified.

## Results

### Transcranial Direct Current Stimulation Reduces the Area and Number of Aβ Plaques in Alzheimer’s Disease Mice

**FIGURE 1:**
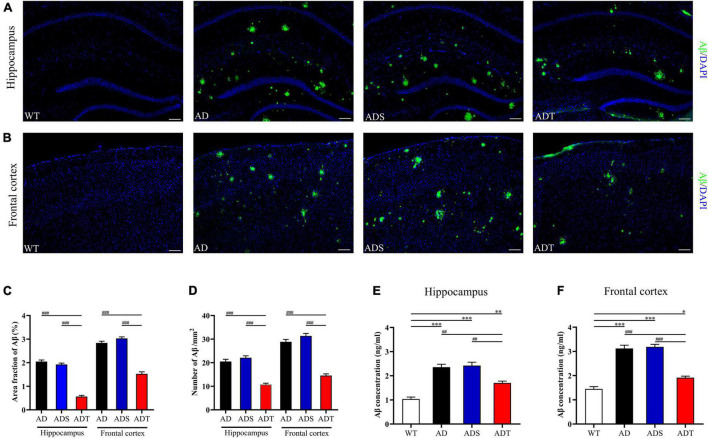
The effect of tDCS on Aβ levels. **(A,B)** Representative images of Aβ (green) in the hippocampus **(A)** and frontal cortex **(B)** in each group. *n* = 5, scale bar = 100 μm. **(C,D)** Area fraction **(C)** and number **(D)** of Aβ plaques in the hippocampus and frontal cortex in each group. *n* = 5. **(E,F)** The concentration of Aβ in the hippocampus **(E)** and frontal cortex **(F)** in each group. *n* = 3. The data are expressed as the mean ± SEM. ^#^, compared with the WT group. *, compared with the ADT group. *P < 0.05, ^##/^**P < 0.005, ^###/^***P < 0.001.

We performed fluorescence staining to observe Aβ plaques in the hippocampus and frontal cortex of mice (Figures 1A,B). Aβ staining was not observed in the WT group. Compared with those in the AD group and ADS group, the area fraction and number of Aβ plaques in the hippocampus and frontal cortex in the ADT group were significantly decreased (*P* < 0.001, Figures 1C,D). An ELISA kit was used to further assess the Aβ concentration in the hippocampus and frontal cortex in each group (Figures 1E,F). Compared with that in the AD group and ADS group, the Aβ concentration in the hippocampus in the ADT group was significantly decreased (*P* < 0.005). Similarly, the Aβ concentration in the frontal cortex was significantly decreased in the ADT group (*P* < 0.001). The concentration of Aβ in the hippocampus and frontal cortex in the AD group and ADS group was significantly higher than that in the WT group (*P* < 0.001), but the concentration of Aβ in the hippocampus (*P* < 0.005, Figure 1E) and frontal cortex (*P* < 0.05, Figure 1F) was also higher in the ADT group than in the WT group. Overall, these results suggest that tDCS can reduce the proportion and quantity of Aβ plaques in the hippocampus and frontal cortex in AD mice.

### Transcranial Direct Current Stimulation Affects the Generation Process of Aβ

**FIGURE 2:**
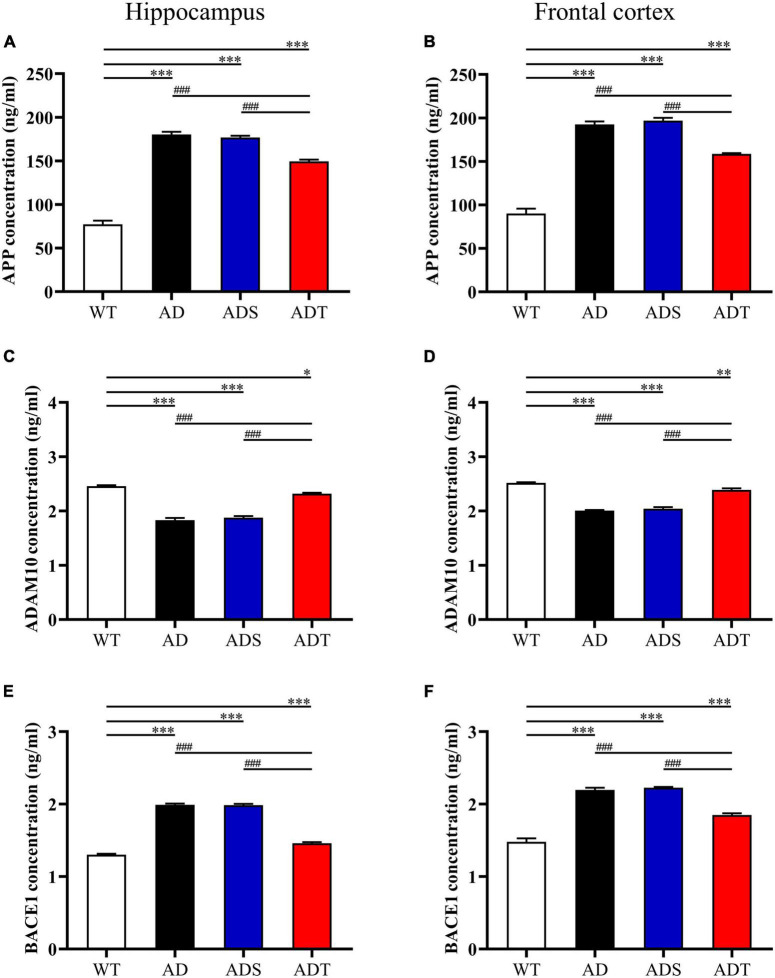
The effect of tDCS on the generation of Aβ. **(A,B)** The concentration of APP in the hippocampus **(A)** and frontal cortex **(B)** in each group. **(C,D)** The concentration of ADAM10 in the hippocampus **(C)** and frontal cortex **(D)** in each group. **(E,F)** The concentration of BACE1 in the hippocampus **(E)** and frontal cortex **(F)** in each group. *n* = 3. The data are expressed as the mean ± SEM. ^#^, compared with the WT group. *, compared with the ADT group. *P < 0.05, **P < 0.005, ^###/^***P < 0.001.

Transcranial direct current stimulation (tDCS) decreased Aβ levels in AD mice, prompting us to further explore the mechanism by which tDCS affects Aβ production. The enzymes α-, β- and γ-secretase cleave APP to produce Aβ peptides (Apatiga-Perez et al., 2021). Thus, we used ELISA to measure the concentrations of APP, ADAM10 and BACE1 in the hippocampus and frontal cortex in mice from each group (Figure 2). APP concentrations in the hippocampus and frontal cortex were higher in the AD group, ADS group and ADT group than in the WT group (*P* < 0.001). Nevertheless, after tDCS, the concentration of APP in the ADT group was very significantly decreased compared with that in the AD group and ADS group (*P* < 0.001) (Figures 2A,B). ADAM10 is a canonical α-secretase that catalyzes non-amyloidogenic cleavage of APP (Seegar et al., 2017). After tDCS, the concentration of ADAM10 in the hippocampus and frontal cortex in the ADT group was significantly higher than that in the AD group and ADS group (*P* < 0.001) (Figures 2C,D). The concentration of ADAM10 in the hippocampus and frontal cortex in the AD group and ADS group was also significantly lower than that in the WT group (*P* < 0.001). Although ADAM10 expression in the hippocampus and frontal cortex in the ADT group was higher than that in the AD group and ADS group, it was still lower than that in the WT group (*P* < 0.05). BACE1, also known as amyloid precursor β-decompose enzyme 1, is an important active molecule involved in Aβ production (Vassar et al., 1999). We compared the concentration of BACE1 in the hippocampus and frontal cortex in mice from each group (Figures 2E,F). Compared with that in the WT group, the concentration of BACE1 in the hippocampus and frontal cortex in the AD group, ADS group and ADT group was higher (*P* < 0.001); however, compared with that in the AD group and ADS group, the concentration of BACE1 in the hippocampus and frontal cortex in the ADT group was significantly decreased (*P* < 0.001). Overall, tDCS reduced Aβ production in AD mice by reducing APP and BACE1 levels and increasing the level of ADAM10.

### Transcranial Direct Current Stimulation Improves Neurovascular Unit Function in Alzheimer’s Disease Mice

**FIGURE 3:**
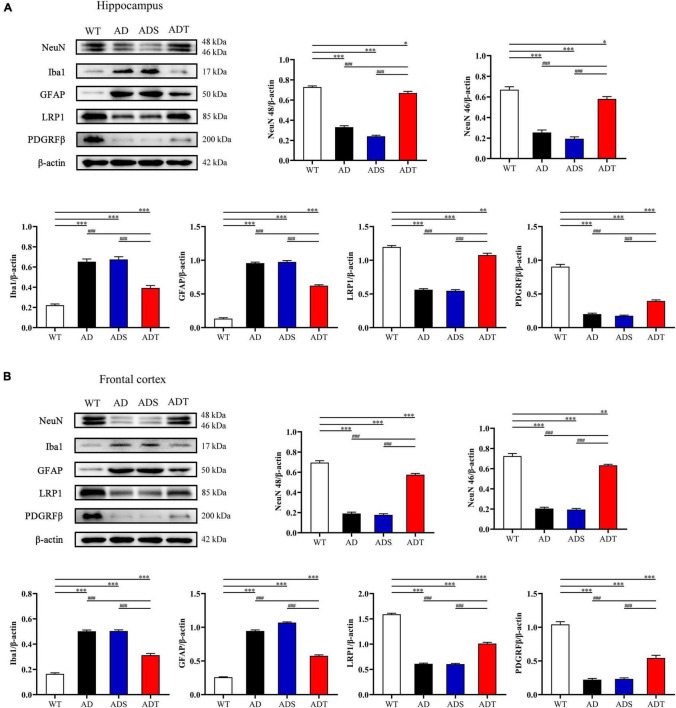
The effect of tDCS on the main components of the NVU. **(A,B)** Representative images and statistical analysis of NeuN, Iba1, GFAP, LRP1 and PDGRFβ expression in the hippocampus **(A)** and frontal cortex **(B)** in each group. *n* = 3. The data are expressed as the mean ± SEM. ^#^, compared with the WT group. *, compared with the ADT group. *P < 0.05, **P < 0.005, ^###/^***P < 0.001.

NVU dysfunction is an important pathogenic process in AD, and the functional integrity of the NVU is crucial for normal neuronal and synaptic function (Zlokovic, 2010). We performed WB to measure the levels of important components of the NVU, such as NeuN, GFAP, ionized calcium-binding adaptor molecule 1 (Iba1), lipoprotein receptor-related protein 1 (LRP1), and the platelet-derived growth factor receptor β (PDGRFβ), in the hippocampus and frontal cortex in mice from each group (Figure 3). NeuN is a common marker of neurons (Wolf et al., 1996). After two weeks of tDCS, the expression of NeuN 46 and NeuN 48 in the hippocampus and frontal cortex in the ADT group were significantly higher than those in the AD group and ADS group (*P* < 0.001), and the expression of NeuN 46 and NeuN 48 in the hippocampus and frontal cortex in the AD group and ADS group were significantly lower than those in the WT group (*P* < 0.001). While the expression of NeuN 46 and NeuN 48 in the ADT group was also significantly different from those in the WT group, they were not as low as those in the AD group and ADS group. Iba1 is a microglial marker that is abnormally expressed in the pathological process of AD (Kirabali et al., 2020; Sahu et al., 2021). Compared with those in the WT group and ADT group, the levels of Iba1 in the hippocampus and frontal cortex in the AD group and ADS group were significantly higher (*P* < 0.001). The expression of Iba1 in the hippocampus and frontal cortex in the ADT group was also higher than that in the WT group (*P* < 0.001). GFAP, which is considered a marker of mature astrocytes, is expressed in normal subjects and is overexpressed in AD patients (Carter et al., 2019). Compared with those in the WT group, the expression of GFAP in the hippocampus and frontal cortex in the AD group, ADS group and ADT group were significantly higher (*P* < 0.001), but the expression of GFAP in the hippocampus and frontal cortex in the ADT group were significantly lower than those in the AD group and ADS group (*P* < 0.001). LRP1 has a neuroprotective effect and protects the blood-brain barrier, but LRP1 expression often decreases with the progression of AD (Wang et al., 2021). The expression of LRP1 in the hippocampus and frontal cortex in the AD group and ADS group were significantly lower than those in the ADT group and WT group (*P* < 0.001). The expression of LRP1 in the hippocampus and frontal cortex in the ADT group was higher than those in the AD group and ADS group but also significantly lower than those in the WT group, in the hippocampus (*P* < 0.005) and frontal cortex (*P* < 0.001). PDGFRβ is thought to be a major cell surface marker defining the pericytes, and pericytes have mainly been associated with stabilization and hemodynamic processes of blood vessels (Song et al., 2005). Compared with the WT group, The expression of PDGFRβ in the hippocampus and frontal cortex in the AD group, ADS group and ADT group were significantly lower (*P* < 0.001), while compared with the AD group and ADS group, the expression of PDGFRβ in the ADT group were significantly increased (*P* < 0.001). In conclusion, tDCS has a protective effect on neurons and exerts beneficial effects on the NVU.

### Transcranial Direct Current Stimulation Protects Blood-Brain Barrier Integrity in Alzheimer’s Disease Mice

**FIGURE 4:**
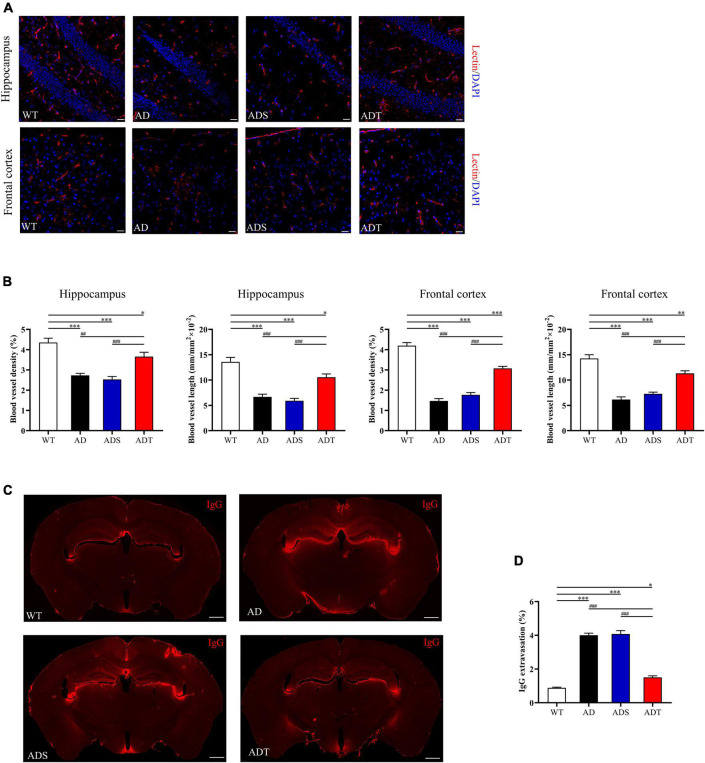
The effects of tDCS on vessel morphology and blood-brain barrier permeability. **(A,B)** Representative images of vascular (red) **(A)** and blood vessel density and blood vessel length **(B)** in the hippocampus and frontal cortex in each group. *n* = 5, scale bar = 100 μm. **(C,D)** Representative images **(C)** and comparisons **(D)** of IgG staining in the hippocampus and frontal cortex of each group. *n* = 5, scale bar = 400 μm. The data are expressed as the mean ± SEM. ^#^, compared with the WT group. *, compared with the ADT group. *P < 0.05, ^##/^**P < 0.005, ^###/^***P < 0.001.

Next, we performed lectin staining and immunofluorescence staining on IgG, occludin, and AQP4 in the blood-brain barrier. We observed the morphology of blood vessels in the hippocampus and frontal cortex by lectin staining (red) (Figure 4A) and analyzed blood vessel density and blood vessel length (Figure 4B). In the hippocampus, the blood vessel density and blood vessel length in the AD group, ADS group and ADT group were significantly lower than that in the WT group (AD group and ADS group: *P* < 0.001; ADT group: *P* < 0.05). However, the blood vessel density and blood vessel length in the ADT group were significantly higher than that in the AD group and ADS group (AD group: *P* < 0.005; ADS group: *P* < 0.001). In the frontal cortex, the blood vessel density and blood vessel length in the AD group and ADS group were significantly lower than that in the WT group and ADT group (*P* < 0.001), although the blood vessel density and blood vessel length in the ADT group were significantly higher than that in the AD group and the ADS group, it was still significantly lower than that in WT group (*P* < 0.005). In the hippocampus and frontal cortex of the WT group and ADT group, the blood vessels had distinct morphology, while part of the vascular area was missing in the AD group and the ADS group, and the vessel morphology was significantly changed.

We showed the representative results of IgG extravasation fluorescence staining on brain sections of WT group, AD group, ADS group and ADT group (Figure 4C), and a semi-quantitative analysis on the percentage of IgG extravasation in brain sections of each group (Figure 4D). It can be seen from the figure that the percentage of IgG extravasation in the AD group and the ADS group was significantly higher than that in the ADT group and WT group (*P* < 0.001). However, compared with the WT group, the percentage of IgG extravasation in the ADT group was also significantly higher (*P* < 0.05).

**FIGURE 5:**
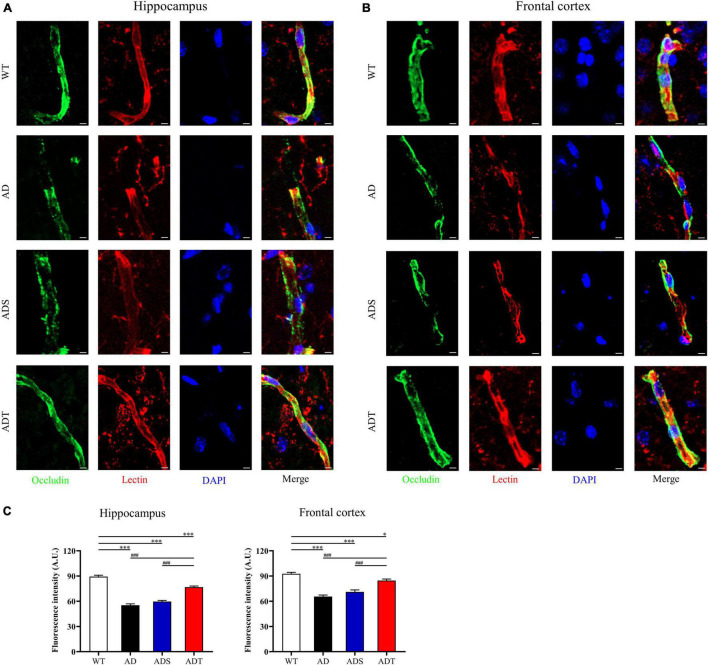
The effect of tDCS on endothelial cell-specific junction protein. **(A–C)** Representative images **(A,B)** and statistical analysis **(C)** of immunofluorescence staining for occludin (green) and lectin staining (red) in the hippocampus and frontal cortex in each group. *n* = 5, scale bar = 2 μm. The data are expressed as the mean ± SEM. ^#^, compared with the WT group. *, compared with the ADT group. *P < 0.05, ^###/^***P < 0.001.

We further analyzed the relationship of occludin and lectin in the hippocampus and frontal cortex of each group (Figures 5A,B, occludin: green; lectin: red and DAPI: blue), as well as semi-quantitative analysis of the fluorescence intensity of occludin in the hippocampus and frontal cortex (Figure 5C). The fluorescence intensity of occludin in the AD group and ADS group in the hippocampus was significantly lower than that in the WT group and ADT group (*P* < 0.001). Compared with the AD group and ADS group, the fluorescence intensity of occludin in the ADT group increased significantly, but it was still significantly lower than that in the WT group (*P* < 0.001). There was the same trend in the frontal cortex.

**FIGURE 6:**
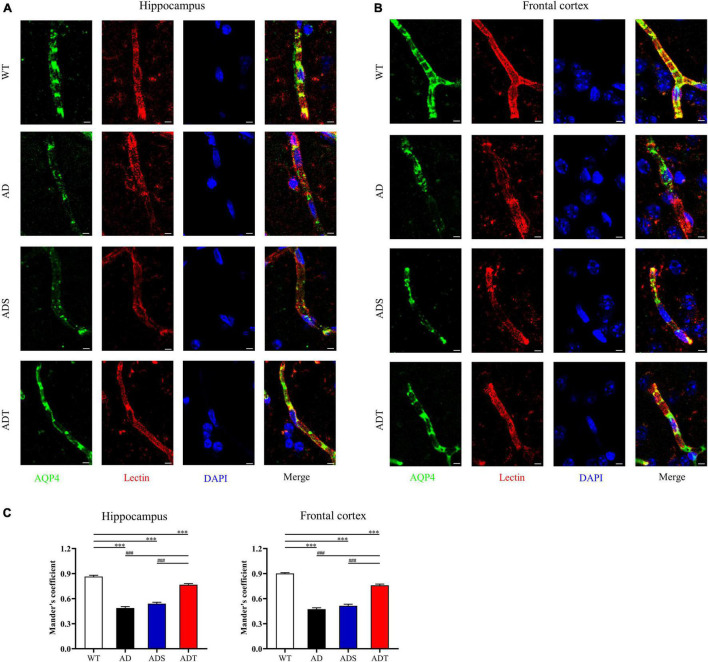
The effect of tDCS on astrocyte foot processes with blood vessels. **(A–C)** Representative images **(A,B)** and statistical analysis **(C)** of immunofluorescence staining for AQP4 (green) and lectin staining (red) in the hippocampus and frontal cortex in each group. *n* = 5, scale bar = 2 μm. The data are expressed as the mean ± SEM. ^#^, compared with the WT group. *, compared with the ADT group. ^###/^***P < 0.001.

To observe the connection between astrocytes and blood vessels more clearly, we analyzed the relationship of AQP4 and lectin in the hippocampus and frontal cortex of each group (Figures 6A,B, AQP4: green; lectin: red and DAPI: blue). The Mander’s colocalization coefficient of AQP4 and lectin in the hippocampus and frontal cortex in the AD group, ADS group and ADT group was significantly lower than that in the WT group (*P* < 0.001). However, the Mander’s colocalization coefficient of the ADT group was significantly higher than the AD group and ADS group (*P* < 0.001) (Figure 6C). In APP/PS1 mice, blood vessel density was decreased, IgG extravasation was increased, the expression of the occludin was decreased, and coverage of astrocyte foot processes with blood vessels was reduced, these results suggested that the blood-brain barrier was impaired in APP/PS1 mice, whereas tDCS rescued this damage.

### Transcranial Direct Current Stimulation Affects Different Types of Astrocytes and Blood Vessels

**FIGURE 7:**
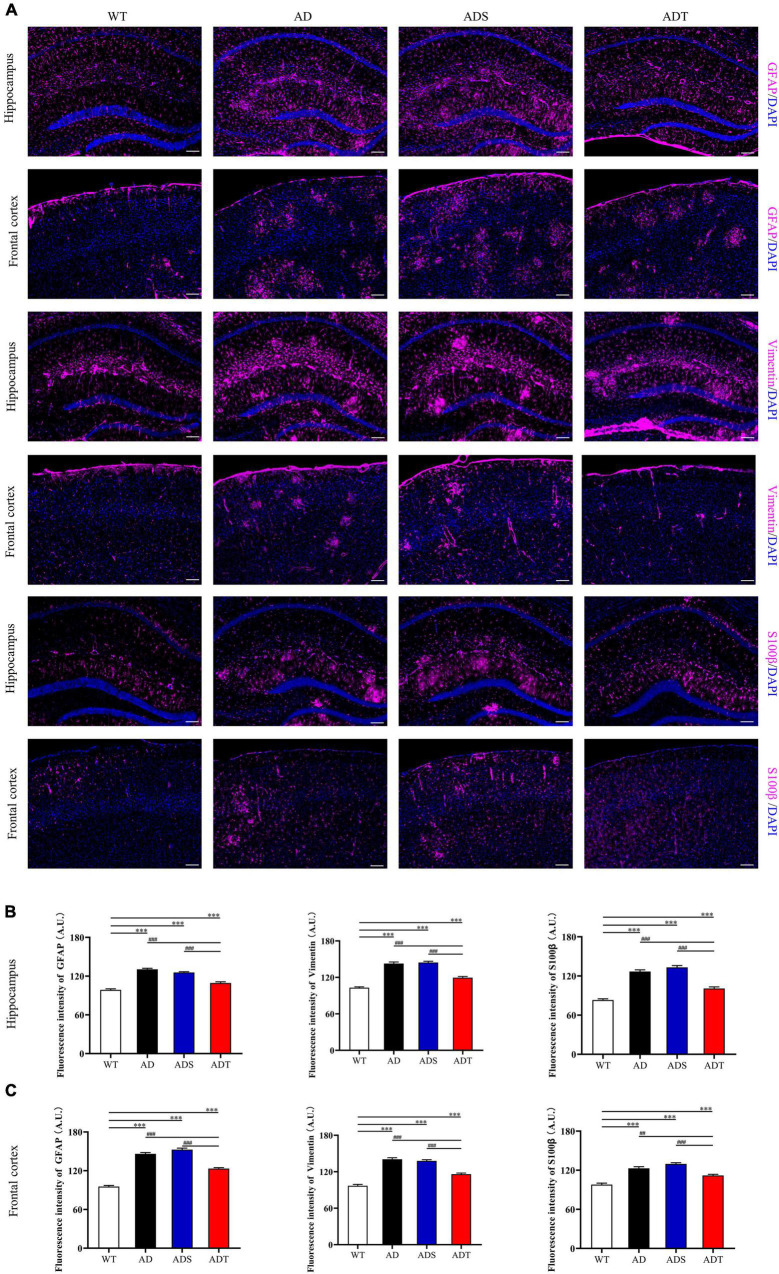
The effect of tDCS on different astrocytes. **(A–C)** Representative images **(A)** and statistical analysis **(B,C)** of immunofluorescence staining for GFAP (purple), Vimentin (purple), and S100β (purple) in the hippocampus and frontal cortex in each group. *n* = 5, Scale bar = 100 μm. The data are expressed as the mean ± SEM. ^#^, compared with the WT group. *, compared with the ADT group. ^##^*P* < 0.005, ^###/^***P < 0.001.

We further explored the effects of tDCS on different types of astrocytes in the hippocampus and frontal cortex in AD mice. We performed immunofluorescence staining for GFAP, a marker of mature astrocyte, Vimentin, a marker of astrocyte precursors and S100β, a marker of vascular astrocytes (Figure 7A). We measured the fluorescence intensity of different types of astrocytes in fluorescence staining images (Figures 7B,C). Consistent with the trend of WB results of GFAP protein, the expression of GFAP-positive cells in the hippocampus and prefrontal cortex of the AD group and ADS group was higher than that of the ADT group and WT group (*P* < 0.001). Although the ADT group had less expression of GFAP-positive cells in the hippocampus and frontal cortex than the AD and ADS groups, the ADT group had significantly more expression of these cells in these regions than the WT group (*P* < 0.001). The expression of Vimentin-positive cells and S100β-positive cells showed the same trend.

**FIGURE 8:**
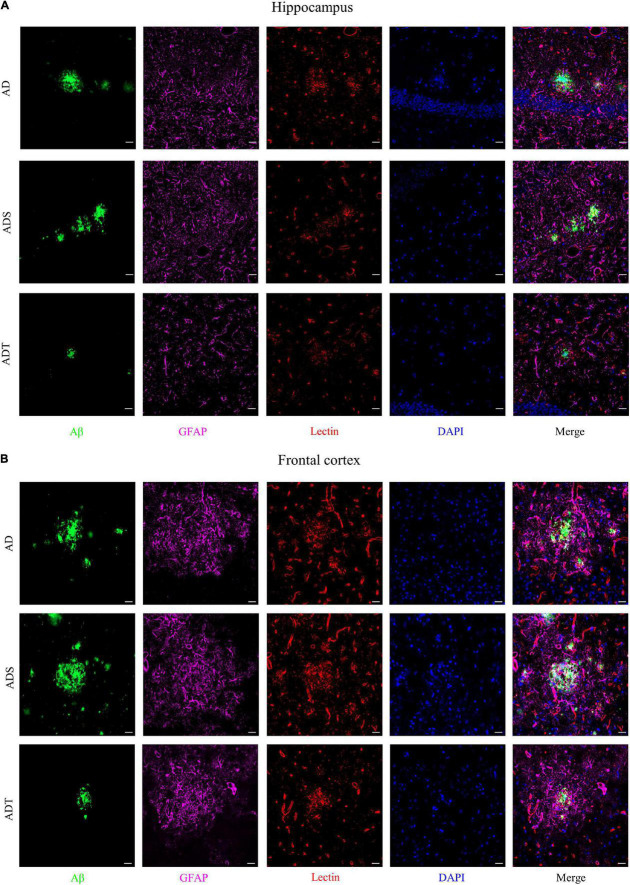
The effect of tDCS on GFAP expression and blood vessels. **(A,B)** Representative images of immunofluorescence staining for Aβ (green) and GFAP (purple) and lectin staining (red) in the hippocampus **(A)** and frontal cortex **(B)** in each group. *n* = 5, Scale bar = 20 μm.

**FIGURE 9:**
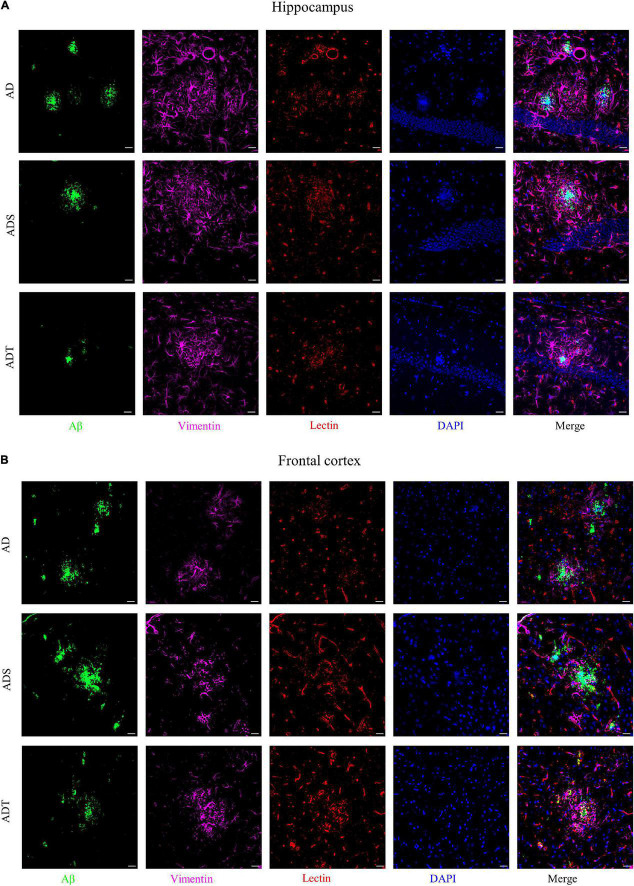
The effect of tDCS on Vimentin expression and blood vessels. **(A,B)** Representative images of immunofluorescence staining for Aβ (green) and Vimentin (purple) and lectin staining (red) in the hippocampus **(A)** and frontal cortex **(B)** in each group. *n* = 5, Scale bar = 20 μm.

**FIGURE 10:**
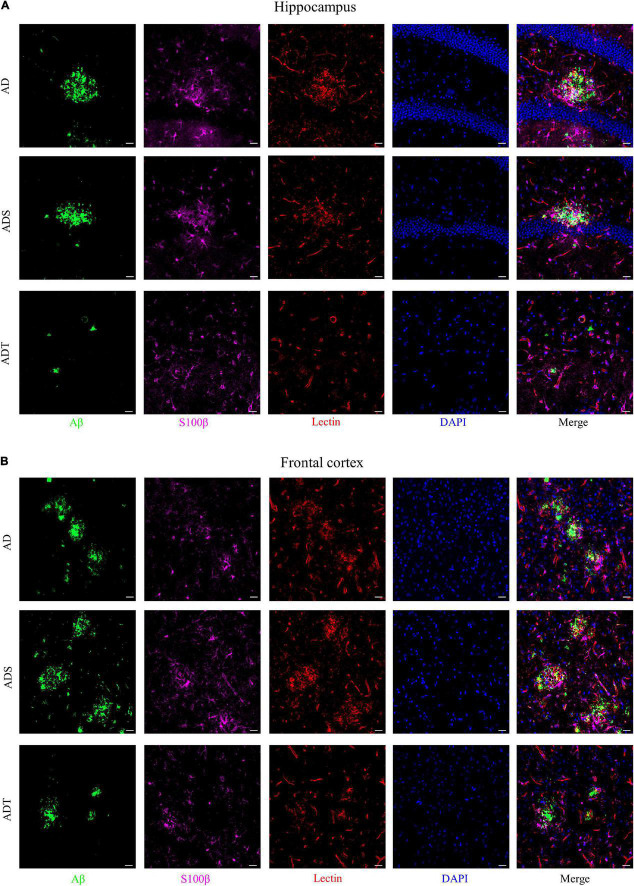
The effect of tDCS on S100β expression and blood vessels. **(A,B)** Representative images of immunofluorescence staining for Aβ (green) and S100β (purple) and lectin staining (red) in the hippocampus **(A)** and frontal cortex **(B)** in each group. *n* = 5, Scale bar = 20 μm.

Immunofluorescence staining of Aβ, astrocytes, astrocyte precursor cells, and lectin staining [Figure 8 (Aβ, GFAP, and lectin), Figure 9 (Aβ, Vimentin, and lectin), Figure 10 (Aβ, S100β, and lectin)] further revealed the relationship among plaques, astrocytes, and the vascular system after tDCS. There was a large number of plaques in the hippocampus and frontal cortex in the AD and ADS groups. GFAP, Vimentin and S100β were overexpressed, and astrocyte endfeet wrapped around the Aβ plaques. The expression of blood vessel markers around Aβ plaques was also increased, and S100β was expressed on most of the blood vessels. In the ADT group, the number and area of Aβ plaques were significantly decreased, and the increase in the number of astrocytes was alleviated. In the ADT group, the expression of blood vessel markers surrounding Aβ plaques was decreased, and the blood vessels were partially covered by astrocyte endfeet.

## Discussion

In this work, the effect of tDCS on the NVU, especially the blood-brain barrier and astrocytes, in APP/PS1 mice with preclinical AD was investigated. Two months after APP/PS1 mice in the preclinical stage were treated with tDCS, Aβ levels were significantly reduced, and Aβ metabolism and the expression of NVU components were significantly improved in the hippocampus and frontal cortex. This finding indicated that the effect of tDCS on preclinical AD persists for at least two months and that tDCS affects Aβ metabolism and the NVU. Analysis of the blood-brain barrier function and the degree of astrocyte differentiation suggested that the benefits of tDCS seem to be closely related to astrocytes and blood vessels.

Abnormal deposition of Aβ is a well-known pathological hallmark of AD, and Aβ neurotoxicity can cause synaptic damage, oxidative stress, inflammation, neuronal degeneration, and damage to the blood-brain barrier (Deane et al., 2004; Di Carlo, 2010; Selkoe and Hardy, 2016; Alzheimer’s Association, 2021). A reduction in Aβ levels has been used as an indicator of the ability of different treatments to ameliorate AD for many years. The frontal cortex and hippocampus are important areas related to cognition and memory (Squire, 1992; Duncan and Owen, 2000) and the main areas of Aβ deposition (Xiong et al., 2011; Sepulcre et al., 2013). Consistent with other studies (Zhou et al., 2017), Aβ was found to be widely expressed in the frontal cortex and hippocampus in APP/PS1 mice. In this study, immunofluorescence staining and ELISA revealed that Aβ levels in the frontal cortex and hippocampus were significantly reduced in APP/PS1 mice in the preclinical stage two months after tDCS (Figure 1). A decrease in Aβ levels has been reported in APP/PS1 mice in the preclinical stage after tDCS (Luo et al., 2020a). This finding shows that the effect of tDCS in reducing Aβ levels can persist for at least two months and that tDCS is beneficial to the early treatment of AD.

An imbalance in the production and clearance of Aβ is responsible for the abnormal aggregation of Aβ, as it results in the amyloidogenic processing of APP. APP is cleaved by β-secretase and γ-secretase to produce Aβ via the amyloidogenic pathway or by α-secretase via the non-amyloidogenic pathway (Di Carlo, 2010; Apatiga-Perez et al., 2021). Changes in the levels of proteins related to Aβ metabolism, including an increase in APP and β-secretase levels and a decrease in α-secretase levels, have been widely reported in AD patients and animal models (Xiong et al., 2011; Lichtenthaler et al., 2021). BACE1 and ADAM10 are typical β-secretase and α-secretase, respectively (Lichtenthaler et al., 2021). Consistent with previous studies (Sun et al., 2019; Zhang et al., 2019; Long et al., 2021), APP and BACE1 protein levels were significantly increased and ADAM10 protein levels were significantly decreased in APP/PS1 mice two months after tDCS, APP protein and BACE1 protein levels were significantly decreased, and ADAM10 protein levels were increased in APP/PS1 mice (Figure 2). This finding indicates that tDCS is involved in the metabolism of Aβ, altering the amyloidogenic and non-amyloidogenic processing of APP to reduce the level of Aβ and that this effect can persist for at least 2 months.

The NVU is a functional structure composed of neurons, glial cells, and cerebral blood vessels. The clearance of Aβ is closely related to the NVU, especially glial cells and blood vessels (Zlokovic, 2011). Astrocytes and microglia are the two most important types of glial cells in AD. Astrocytes contribute to the clearance of Aβ by expressing Aβ-degrading enzymes and related receptors, and microglia are involved in phagocytosis in the normal brain (Apatiga-Perez et al., 2021). However, under pathological conditions, these cells are overactivated, causing inflammation and increasing the deposition of Aβ (Kwon and Koh, 2020; Zhao et al., 2021). In the cerebrovascular system, the transporter LRP1 passes through the blood-brain barrier to transport Aβ from around blood vessels to the circulating blood, thus participating in the clearance of Aβ (Deane et al., 2004; Iadecola, 2017). A study on postmortem cortical and hippocampal samples from AD patients and non-demented elderly people showed that all the main components of the NVU show obvious detrimental changes (Kirabali et al., 2020). In our research, similar results were observed. The levels of a neuronal marker (NeuN), a transporter protein (LRP1) and a pericyte marker (PDGRFβ) were decreased, and the levels of an astrocyte marker (GFAP) and microglial marker (Iba1) were increased in the frontal cortex and hippocampus in APP/PS1 mice. These results suggest that NVU function and Aβ clearance are blocked in APP/PS1 mice. In APP/PS1 mice treated with tDCS, the levels of NeuN and LRP1 were increased, and the levels of GFAP and Iba1 were decreased (Figure 3). This finding shows that tDCS can improve NVU function in APP/PS1 mice and indirectly improve the clearance of Aβ. How does tDCS improve the function of the NVU? Recent studies on the effects of tDCS on astrocytes and cerebral blood vessels have revealed the potential connection.

Transcranial direct current stimulation (tDCS**)** may effectively alleviate AD by altering astrocytes and blood vessels. In APP/PS1 mice, there were a large number of GFAP-positive cells and blood vessels around Aβ peptides in the cortex and hippocampus, implying a close connection between astrocytes and blood vessels (Figure 8). Astrocytes and neurons wrap brain capillary endothelial cells to form the blood-brain barrier, which is the main mechanism through which Aβ is cleared (Zhao et al., 2021). In AD, the characteristic of the impaired blood-brain barrier has been widely reported (Kirabali et al., 2020). Consistent with previous studies (Janota et al., 2015), impaired blood-brain barrier occurred in the cortex and hippocampus of APP/PS1 mice, including increased vascular permeability (Figure 4), decreased endothelial junction protein expression level (Figure 5), and decreased coverage of astrocyte foot processes with blood vessels (Figure 6). Recently, researchers have proposed that the blood-brain barrier is mediated by astrocytes in AD. The effect of astrocytes on blood vessels is bidirectional. Under normal circumstances, astrocytes release specific protective factors, including vascular endothelial growth factor (VEGF), insulin-like growth factor-1, glial cell-derived neurotrophic factor and angiopoietin-1, to maintain normal blood-brain barrier function. However, under pathological conditions, such as Aβ stimulation, reactive astrocytes secrete vascular permeability factors and proinflammatory factors, including matrix metalloproteinase-9, nitric oxide, apolipoprotein E-4, interleukin-6, and tumor necrosis factor-α, which aggravate or cause blood-brain barrier damage and inflammation (Abbott et al., 2006; Apatiga-Perez et al., 2021; Zhao et al., 2021). Our study showed that tDCS reduced astrocyte levels and improved blood-brain barrier function (Figures 3–6). However, tDCS has been reported to temporarily increase the number of glial cells and the permeability of the blood-brain barrier and later decrease the number of glial cells, normalize the permeability of the blood-brain barrier, and increase the protein and mRNA levels of VEGF and interleukin-8 (Bai et al., 2011; Rueger et al., 2012; Cancel et al., 2018; Bahr-Hosseini and Bikson, 2021). These findings indicate that the effect of tDCS is compensatory. The early increase in the number of astrocytes may promote the permeability of the blood-brain barrier after tDCS, accelerate the clearance of Aβ, reduce neurotoxicity, promote a decrease in the number of astrocytes, increase the expression of blood vessel-related factors, and finally normalize the blood-brain barrier function. This seems to explain why compared with untreated APP/PS1 mice, tDCS-treated APP/PS1 mice exhibited reduced GFAP level (Figure 3), decreased vascular permeability (Figure 4), increased endothelial junction protein expression level (Figure 5), increased coverage of astrocyte foot processes with blood vessels (Figure 6).

Transcranial direct current stimulation **(**tDCS) affects not only mature astrocytes but also astrocyte precursors. Vimentin is a marker of astrocyte precursor cells, and its expression can determine the expression of GFAP (Manzano et al., 2007; Bramanti et al., 2010). S100β labels subtypes of mature astrocytes that are encapsulated by blood vessels (Deloulme et al., 2004; Wang and Bordey, 2008). Vimentin and S100 levels increase during the course of AD (Sheng et al., 1996; Olsen et al., 2018). Consistent with the change in the number of mature astrocytes, tDCS reduced Vimentin and S100β protein levels in the frontal cortex and hippocampus in APP/PS1 mice. It seems that astrocyte precursor cells are also involved in the effect of tDCS. This implies that tDCS also has an effect on the differentiation of astrocytes. Similarly, Vimentin and S100β expression are high and there are many blood vessels around Aβ, Aβ may stimulate the proliferation and regeneration of astrocytes. This indicates that differential differentiation of astrocytes is closely related to blood vessels. However, it should be noted that S100β also labels oligodendrocyte precursor cells (Deloulme et al., 2004). Whether the effect of tDCS is related to oligodendrocyte precursor cells is worthy of further study.

Transcranial direct current stimulation **(**tDCS) exerts marked effects in APP/PS1 mice in the preclinical stage. However, our research has limitations, and further research is still needed. The specific effect of tDCS on astrocytes and blood vessels needs to be further verified by studying proinflammatory factors and vascular-related factors. In the future, we can combine imaging technologies to study the effect of tDCS on astroglia and blood vessels, especially the differentiation of astrocytes. Oligodendrocyte precursor cells are involved in memory consolidation, and the effect of tDCS on oligodendrocyte precursor cells and blood vessels is also a focus of research. Microglia-related neuroinflammation is closely related to neurovascular coupling in AD (Duncombe et al., 2016), and whether tDCS can induce immune-inflammatory changes in AD is also worth exploring.

## Conclusion

Our results indicate that Aβ plaques promote the proliferation of astrocytes and impair the blood-brain barrier in AD. Transcranial direct current stimulation may promote the clearance of Aβ plaques by regulating the proliferation of astrocytes and the NVU and further alleviate AD pathology. Our findings provide promising evidence for the effectiveness of tDCS in alleviating preclinical AD pathology.

## Data Availability Statement

The original contributions presented in the study are included in the article/supplementary material, further inquiries can be directed to the corresponding authors.

## Ethics Statement

The animal study was reviewed and approved by the Experimental Animal Welfare and Ethics Committee of Army Medical University.

## Author Contributions

YL, GYW, and HW designed the study. YL, HY, XY, and YW performed the experiments. HY, GLW, and GYW participated in data analyses. YL, HY, and XW drafted the manuscript. XT, YX, and HW reviewed and modified the manuscript. All authors contributed to the article and agreed to be accountable for all aspects of the work.

## Conflict of Interest

The authors declare that the research was conducted in the absence of any commercial or financial relationships that could be construed as a potential conflict of interest.

## Publisher’s Note

All claims expressed in this article are solely those of the authors and do not necessarily represent those of their affiliated organizations, or those of the publisher, the editors and the reviewers. Any product that may be evaluated in this article, or claim that may be made by its manufacturer, is not guaranteed or endorsed by the publisher.
